# Supplementary material for: Precision long-read metagenomics sequencing for food safety by detection and assembly of Shiga toxin-producing Escherichia coli in irrigation water
Source: PLoS One. 2021 Jan 14;16(1):e0245172. doi: 10.1371/journal.pone.0245172 (PMC7808635; doi:10.1371/journal.pone.0245172)
Supplement: S1 Table — (DOCX) [file pone.0245172.s001.docx]

**S1 Table**. qPCR primers and probes used in this study.

|  | **Genbank** | **Primers and Probe** |
| --- | --- | --- |
| *stx1* | M19473 | F -GTGGCATTAATACTGAATTGTCATCA  R – GCGTAATCCCACGGACTCTTC  Cy5 -TGATGAGTTTCCTTCTATGTG TCCGGCAGAT- BHQ2 |
| *stx2* | X07865 | F – GATGTTTATGGCGGTTTTATTTGC  R – TGGAAAACTCAATTTTACCTTTAGCA  TAMRA -TCTGTTAATGCAATGGCGGCGGAT T- BHQ2 |
| *wzy* | AF061251    AF305917 | F – CTCGATAAATTGCGCATTCTATTC  R – CAATACGGAGAGAAAAGGACCAA  6FAM - ACTTAGTGGCTGGGAATGCATCGGC – BHQ1 |
