# Supplementary material for: Precision long-read metagenomics sequencing for food safety by detection and assembly of Shiga toxin-producing Escherichia coli in irrigation water
Source: PLoS One. 2021 Jan 14;16(1):e0245172. doi: 10.1371/journal.pone.0245172 (PMC7808635; doi:10.1371/journal.pone.0245172)
Supplement: S2 Table — (DOCX) [file pone.0245172.s002.docx]

**S2 Table**. Oxford Nanopore sequencing output.

| **Sample** | **DNA concentration (ng/ul)** | **DNA extracted (ug)** | **ng/rxn^a^** | **Total Output (Gb)** | **Reads (M)** | **Total Yield (Gb)** | **Average Sequence Length (kb)** | **Max Sequence Length (bp)** |
| --- | --- | --- | --- | --- | --- | --- | --- | --- |
| Water | 69.2 | 5.54 | 193.2 | 20.94 | 5.75 | 16.9 | 3.8 | 132,403 |
| Water+Ecoli1 | 102 | 8.16 | 338.4 | 18.71 | 4.50 | 14.00 | 4.4 | 220,004 |
| Water+Ecoli2 | 80.2 | 6.42 | 169.2 | 17.19 | 4.19 | 14.7 | 4.3 | 159,036 |
| Water+Ecoli3 | 82.2 | 6.58 | 204.0 | 23.18 | 6.66 | 20.9 | 3.6 | 132,038 |
| Water+Ecoli4 | 74.4 | 5.95 | 186.0 | 24.30 | 5.23 | 21.6 | 4.8 | 155,640 |
| Water+Ecoli5 | 82.2 | 6.58 | 158.4 | 20.04 | 5.28 | 15.7 | 4.0 | 108,437 |
| Water+Ecoli6 | 81.2 | 6.50 | 193.2 | 18.35 | 4.28 | 13.8 | 4.0 | 114,749 |
| Water+Ecoli7 | 79.0 | 6.30 | 204.0 | 12.51 | 2.72 | 8.07 | 4.0 | 126,713 |
| Average |  |  |  | 19.40 | 4.83 | 15.71 |  |  |
| SD |  |  |  | 2.61 | 0.89 | 3.22 |  |  |

SD = standard deviation

^a^total DNA library added to the flow cell
