# Supplementary material for: Precision long-read metagenomics sequencing for food safety by detection and assembly of Shiga toxin-producing Escherichia coli in irrigation water
Source: PLoS One. 2021 Jan 14;16(1):e0245172. doi: 10.1371/journal.pone.0245172 (PMC7808635; doi:10.1371/journal.pone.0245172)
Supplement: S4 Table — (DOCX) [file pone.0245172.s004.docx]

**S4 Table.** MAG fragmented complete or partial genomes recovered from the enriched water sample (representing > 1% abundance in the sample).

| **taxa** | **Contigs id as taxa** | **contigs cumulative size (Mb)** | **Expected genome size (Mb)** | **Coverage X^a^** |
| --- | --- | --- | --- | --- |
| *Klebsiella pneumoniae* | 35 | 6.04 | 5.3 | 1.14 |
| *Enterobacter cloacae* | 167 | 8.23 | 4.9 | 1.68 |
| *Enterobacter sp.* | 13 | 0.24 | 4.8 | 0.05 |
| *Enterobacter kobei* | 11 | 0.71 | 4.9 | 0.15 |
| *Pseudomonas putida* | 214 | 12.35 | 6.3 | 1.96 |
| *Citrobacter freundii* | 21 | 4.45 | 5.4 | 0.83 |
| *Acinetobacter baumannii* | 17 | 4.01 | 3.9 | 1.03 |
| *Enterobacter hormaechei* subsp. *hormachei* | 42 | 0.99 | 4.8 | 0.21 |
| *Enterobacter hormaechei* subsp. *Xiangfangensis* | 11 | 0.21 | 4.8 | 0.04 |

^a^Genomes with coverage >1X are considered as fragmented complete MAG for that specie.
