# Supplementary material for: Precision long-read metagenomics sequencing for food safety by detection and assembly of Shiga toxin-producing Escherichia coli in irrigation water
Source: PLoS One. 2021 Jan 14;16(1):e0245172. doi: 10.1371/journal.pone.0245172 (PMC7808635; doi:10.1371/journal.pone.0245172)
Supplement: S1 Note — (DOCX) [file pone.0245172.s005.docx]

**S1 Note**. Custom python script to extract reads for a desired taxon from WIMP classified nanopore data.

from Bio import SeqIO

import pandas as pd

import re

#---------------------------------------------------------

#Replace the string in parenthesis with your own wimp csv

wimpfile='216244_classification_wimp_v2-v1.csv'

#-------------------------------------------

#Specify your preferred filter id here

id ='Escherichia coli*'

#-------------------------------------

#Replace the string after the equal sign with your own fastq file

data_file = "all.fastq"

#------------------------------------------

#Output file name

outfile= "outindexBC18.fastq"

#---------------------------------------------------------

df = pd.read_csv(wimpfile)

print "Indexing reads from datafile"

rec= SeqIO.index(data_file,"fastq")

print "Done indexing...Filtering reads from CSV..."

reads = []

for index, row in df.iterrows():

    if re.match( id , row['name']):

        reads.append(row['readid'])

print "Done Filtering...Writing out"

F=open(outfile, "w")

for i in reads:

            if i in rec:

                        SeqIO.write(rec[i], F, "fastq")

F.close()
