# Supplementary material for: Precision long-read metagenomics sequencing for food safety by detection and assembly of Shiga toxin-producing Escherichia coli in irrigation water
Source: PLoS One. 2021 Jan 14;16(1):e0245172. doi: 10.1371/journal.pone.0245172 (PMC7808635; doi:10.1371/journal.pone.0245172)

**S1 Fig.** Comparison of the EDL933 genome of the strain used in this study with assemblies obtained from different EDL933 enrichment spiking levels showing the recovery of the *E. coli* O157:H7 MAG either completely closed or fragmented. Each sample extracted EDL933 matching contigs is laid out in a horizontal track and homologous segments are indicated in the same color and connected across genomes. Respective scales show the sequence coordinates in base pairs. A colored similarity plot is shown for each genome, the height of which is proportional to the level of sequence identity in that region. Contigs boundaries are represented by a red line. A) EDL933 vs all *E. coli* O157 MAGs from the different EDL933 spiking levels. From level Water+Ecoli4 we could not recover a complete fragmented O157 MAG. B) EDL933 vs levels where we could recover a completely closed O157 MAG, including the pO157 plasmid for visualization purposes.

A)


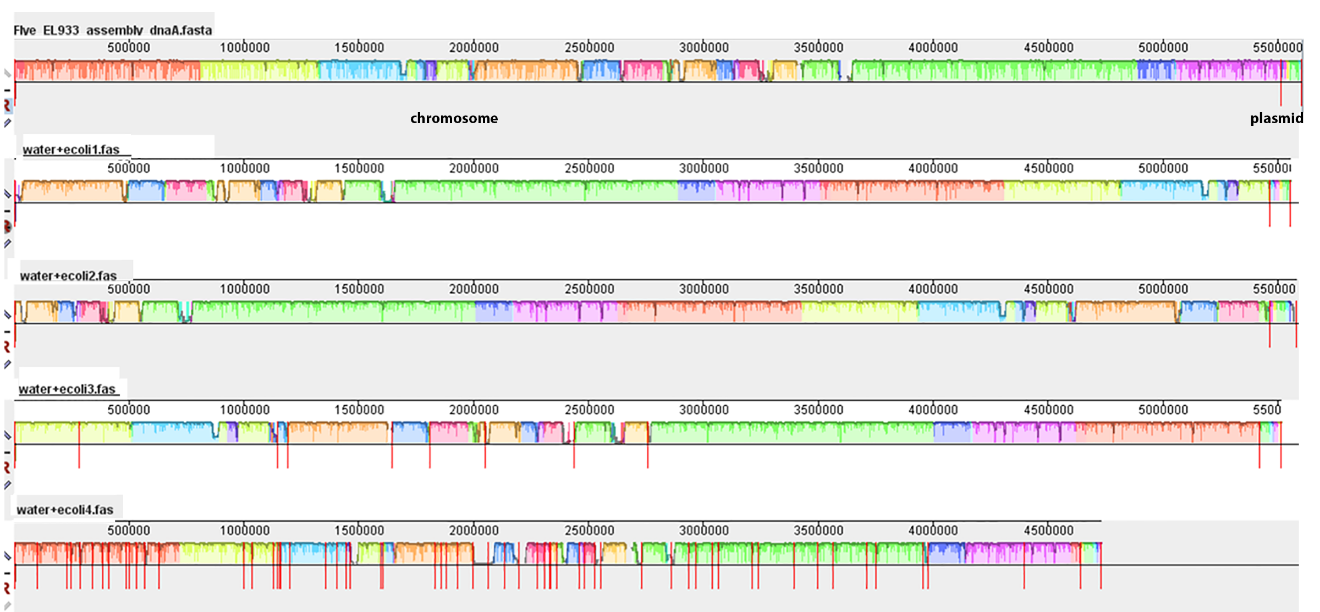


B)


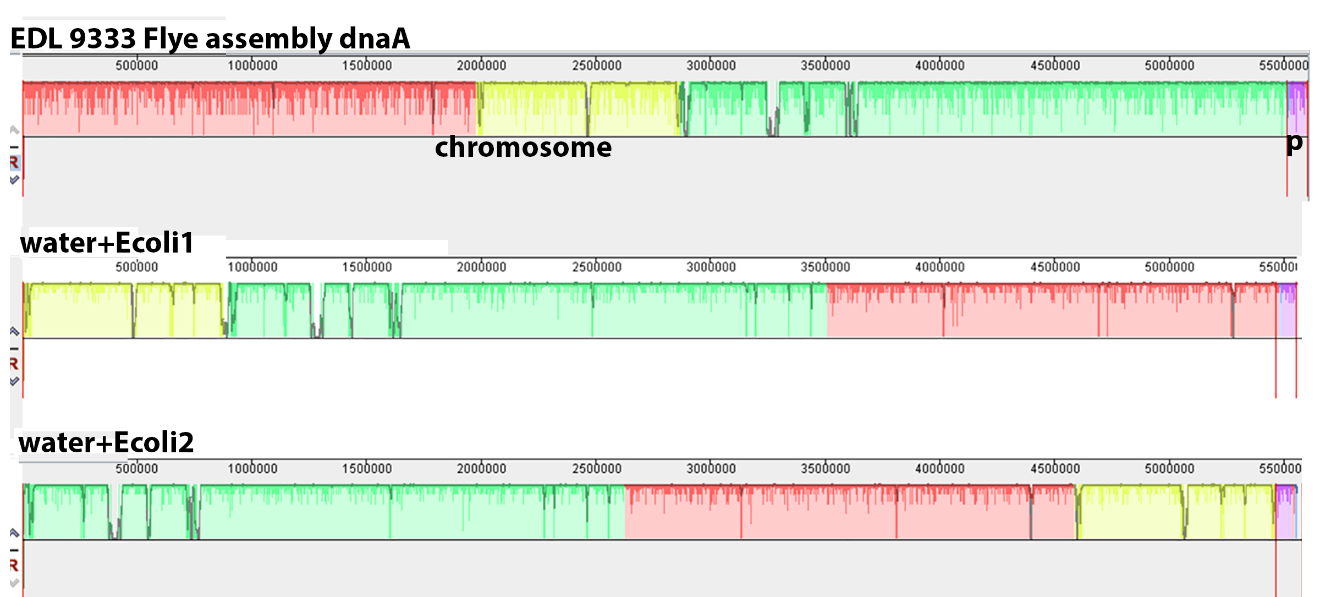

Supplement: S1 Fig — Each sample extracted EDL933_2 matching contigs is laid out in a horizontal track and homologous segments are indicated in the same color and connected across genomes. Respective scales show the sequence coordinates in base pairs. A colored similarity plot is shown for each genome, the height of which is proportional to the level of sequence identity in that region. Contigs boundaries are represented by a red line. A) EDL933_2 vs all E. coli O157 MAGs from the different EDL933_2 spiking levels. From level Water+Ecoli4 we could not recover a complete fragmented O157 MAG. B) EDL933_2 vs levels where we could recover a completely closed O157 MAG, including the pO157 plasmid for visualization purposes. (DOCX) [file pone.0245172.s006.docx]
